# A cross-sectional analysis of the cost and affordability of achieving recommended intakes of non-starchy fruits and vegetables in the capital of Vanuatu

**DOI:** 10.1186/s12889-015-1644-2

**Published:** 2015-03-28

**Authors:** Holly A Jones, Karen E Charlton

**Affiliations:** School of Medicine, Faculty of Science, Medicine and Health, University of Wollongong, Northfields Avenue, Wollongong, NSW Australia

**Keywords:** Fruit and vegetable, Food security, Food affordability, Pacific Islands, Income and expenditure

## Abstract

**Background:**

The low-income Pacific Island nation of Vanuatu is experiencing a double burden of diet-related disease whereby micronutrient deficiencies and underweight occur at the same time as obesity related non-communicable diseases. Increasing intakes of nutrient dense, energy dilute foods such as fruits and vegetables will be important to address this issue. However, reduced access to agricultural land in urban areas provides limited opportunities for traditional subsistence fruit and vegetable production. Set in Port Vila, Vanuatu’s capital and main urban centre, this study aimed to determine the cost and affordability of meeting international recommendations to consume at least 400 g of non-starchy fruits and vegetables (NSFV) per person per day, and assess the adequacy of households’ NSFV expenditure.

**Methods:**

NSFV prices from the 2010 Vanuatu Consumer Price Index (n = 56) were used to determine the minimum monthly cost of purchasing 400 g of local NSFV per person, after accounting for wastage. The 2010 Vanuatu Household Income and Expenditure Survey (n = 578 households) was analysed to determine the proportion of households’ total and food budget required to purchase 400 g of local NSFV for all household members. Household NSFV costs were also compared against actual household expenditure on these items. Consumption of own-produce and gifts received were included within estimates of food expenditure.

**Results:**

The minimum cost of purchasing the recommended amount of local NSFV was 1,486.24 vatu ($16.60 US) per person per month. This level of expenditure would require an average of 9.6% (SD 6.4%) of households’ total budget and 26.3% (SD 25.8%) of their food budget. The poorest households would need to allocate 40.9% (SD 34.3%) of their total food budget to NSFV to purchase recommended amounts of these foods. Twenty-one percent of households recorded sufficient NSFV expenditure while 23.4% recorded less than 10% of the expenditure required to meet the NSFV recommendations.

**Conclusions:**

Achieving recommended intakes of local NSFV in Port Vila is largely unaffordable, and expenditure on these foods was inadequate for most households in Port Vila in 2010. Addressing fruit and vegetable affordability will be an important consideration in prevention of non-communicable diseases in the Pacific region.

## Background

The Pacific Island nation of Vanuatu is currently categorised by the United Nations as a Least Developed Country [1]. Its population of approximately 234,000 is distributed over an archipelago comprising 83 islands, and has an annual growth rate of 2.3% [2]; the third highest in the Pacific [3]. The population is predominantly (98%) Indigenous, known as ni-Vanuatu [4]. Seventy-six percent of the population live in rural areas, although an increasing proportion live in the nation’s two urban areas: Port Vila, the capital of Vanuatu on the island of Efate; and, Luganville on the island of Espiritu Santo [2]. Between 1999 and 2009, Vanuatu’s urban population grew by 42% and close to 20% of the total population now live in Port Vila [2]. Urban residents tend to follow a more westernised way of life, having moved away from the traditional subsistence lifestyle of their rural counterparts [5].

Vanuatu displays a double burden of diet-related disease, which is consistent with other Pacific Islands [6-8]. Children are affected by underweight, stunting and as well as micronutrient deficiencies, with the latter also prevalent in women of reproductive age [9]. Concurrently, risk factors for non-communicable diseases (NCDs) such as overweight and obesity, raised blood glucose and raised blood cholesterol concentrations affect 51, 21 and 37 per cent of the adult population respectively [10]. In 2010, 64 per cent of all deaths in Vanuatu were attributed to diet and lifestyle- related NCDs [11]. The cost of managing NCDs in Vanuatu is straining national financial resources. For example, it is estimated that the cost of effectively managing a single case of diabetes in primary care is 2.2 times higher than the nation’s total per capita expenditure on health [12].

Fruits and vegetables are important in the prevention of both NCDs and micronutrient deficiencies; low intakes are one of the top ten risk factors for global mortality [13]. A population-wide recommendation of at least 400 g (equivalent to 5 servings of 80 g) of fruits and vegetables per person per day is recommended by a World Health Organization (WHO) and Food and Agriculture Organization (FAO) expert panel on diet, nutrition and the prevention of chronic diseases [14]. These recommendations refer to non-starchy fruits and vegetables (NSFV), thus excluding starchy crops such as cassava and potatoes [14]. While Vanuatu does not have its own country-specific dietary guidelines, the *Pacific Guide to Healthy Eating* [15] recommends that Pacific Islanders consume one-third of daily food intake from the ‘*Protective Food Group’*, which includes micronutrient rich items such as leafy vegetables, papaya, citrus, mangoes, pumpkin and bananas but excludes starchy crops such as breadfruit, plantain bananas, cassava and yam. The guide also emphasises dietary variety and priority for local over imported foods.

The majority of the population in Vanuatu falls short of meeting these recommendations, with 62% of adults consuming insufficient NSFV [10]. Insufficient NSFV intakes are prevalent in many developing countries; an analysis of over 196,000 adults in 52 mostly low and middle income countries reported that 78% of adults did not consume the recommended 400 g of NSFV per day [16]. In ten of these countries the urban population was significantly less likely to meet the NSFV recommendations than their rural counterparts, with the converse only observed in one of the 52 countries.

In Vanuatu, the rural population is over seven times more likely than those living in urban areas to consume local foods every day [17], and consumption of imported processed foods is more common in urban areas [17,18]. Throughout the Pacific Islands, eating patterns have shifted from the traditional diet of nutrient dense local foods to one high in energy dense, processed, imported foods [8]. The reduced availability of agricultural land related to urbanisation is thought to have contributed to this issue [19,20] and access to land is considered an indicator of food security in Vanuatu [21]. It is estimated that 30% of urban households in Vanuatu do not have access to land for traditional subsistence agriculture [22] and are thus required to obtain fruits and vegetables through other means, if at all. Population growth is also threatening food security in Vanuatu; between 1983 and 2007, food crop production did not change significantly despite the population size almost doubling [23].

The impact of food insecurity in Vanuatu is evident, with one-fifth (21%) of ni-Vanuatu families nationally reporting that they have had to miss or reduce the size of meals over a twelve-month period due to not having enough food available [21]. In Vanuatu’s urban areas, 29% of families reported adopting this behaviour, including 7% who missed or reduced the size of their meals once or more per month. The mid-term report on Vanuatu’s achievements against the Millennium Development Goals (MDGs) [23] indicates that the nation is unlikely to meet the MDG targets for improving food security, reducing the prevalence of underweight in children and reducing hunger.

Strategies to improve food security in the Pacific Islands identify the importance of local food production and the need for greater promotion of the benefits of these foods [24,25]. Understanding local food cost and affordability will be important to inform the implementation of these strategies and the promotion of healthy diets [26]. Data obtained through the periodic Vanuatu Household Income and Expenditure Survey (HIES) [27], Consumer Price Index (CPI) [28] and UNICEF sentinel price-monitoring [29] contribute to the evidence base on food cost and affordability in Vanuatu. However, they do not compare food costs against recommended food intakes, and therefore more information is required to fully inform food security strategies and the promotion of healthy foods in Vanuatu.

This study aimed to assess the cost and affordability of meeting the recommendations for consumption for NSFV in Port Vila, Vanuatu, and determine the adequacy of households’ expenditure on these foods. Port Vila was selected as the focus of the study as it is the largest urban centre in Vanuatu and is therefore most affected by the threats to food security posed by increasing urbanisation and associated loss of access to land for traditional subsistence agriculture [19].

## Methods

Secondary data analysis was conducted using the 2010 Vanuatu CPI, which provided information on food costs, and the 2010 Vanuatu HIES, which provided information on household expenditure. Both surveys were undertaken by the Vanuatu National Statistics Office (VNSO).

### Consumer price index

The VNSO collects prices of common commodities sold in Port Vila and Luganville as part of its ongoing CPI monitoring activities. In Port Vila, fruit and vegetable prices (per kilogram) are collected on a fortnightly basis from major supermarkets and the central produce markets, however smaller roadside markets are excluded.

In the present study CPI prices for NSFV (i.e. excluding starchy foods such as sweet potato, yam, cassava, taro, plantain banana etc.) in Port Vila during the December 2010 CPI quarter were selected for the analysis. This corresponded with the enumeration period of the 2010 HIES and enabled direct comparison between food costs and household food expenditures.

### Household income and expenditure survey

The Vanuatu HIES was first conducted in 1985, and subsequently in 1998, 2006 and 2010. The 2010 HIES objectives included updating the basket of items collected in the CPI; evaluating social programs and policies; and, collecting key poverty indicator statistics. The survey used a two-stage sampling method where enumeration areas were first identified using probability proportional to size sampling using the 2009 Census [2] as the sample frame. Households were then selected through systematic sampling of lists of households in the enumeration area. Non-private dwellings, such as schools or hospitals were excluded, as were expatriate temporary residents and permanent residents not intending to reside in Vanuatu for at least 12 months.

The 2010 HIES was enumerated over a three month period between October and December 2010. Data collection was performed using a combination of interviewer-administered questionnaires with the household head and daily expenditure diaries completed by all adult household members over a two-week period. Household expenditure was calculated by summing the value of cash purchases, subsistence production, barter or income-in-kind, gifts received and non-consumption expenditure such as payments to government, religious organisations or other Vanuatu households without acquiring any goods or services in return. The value of items such as gifts or subsistence produce was determined by asking participants to estimate the value of the item if it were sold locally. If the value of such items was unknown, a value was imputed by the VNSO based on transactions for the same commodity in the same location.

Within Port Vila, 89.5% of households selected for the HIES provided a complete response, resulting in a sample size of 578 households. Further information on the 2010 HIES methodology is available from the VNSO [27].

### Data analysis

In the present analysis, data were analysed using Microsoft Excel for Mac version 14.3.9 (Microsoft Corporation, Redmond, WA, USA). The Statistical Package for the Social Sciences (SPSS) version 19.0 (SPSS Inc, Chicago IL, USA) was used to assess differences in expenditure according to household size using Analysis of Variance (ANOVA).

The CPI prices were initially examined to identify suspected data entry errors, which were subsequently corrected or excluded from the analysis based on advice from the VNSO. Prices for imported NSFV (e.g. imported tomatoes, lettuce, apples, pears and tinned or frozen NSFV) were excluded to comply with regional recommendations for prioritising consumption of local foods. NSFV primarily used in small amounts as a flavour or garnish were also excluded from the analysis, as these are unlikely to be consumed in amounts equivalent to a serving per day. A Vanuatu Ministry of Health recipe book (unpublished observations) was consulted to confirm typical uses of local produce. Green and mature coconuts were also excluded from the analysis as they were not considered to be a fruit or vegetable.

The remaining NSFV prices were converted into prices per edible 80 g serve using edible portions from the USDA National Nutrient Database for Standard Reference, Release 25 [30]. As information on the edible portion of island cabbage (*Abelmoschus manihot*) was unavailable, it was assumed that there is no wastage for this item.

The minimum cost for an individual to meet the recommended intake of at least 400 g (five serves) of NSFV per day was determined by summing the price per edible 80 g serve of the cheapest two fruits, the cheapest two vegetables and the next cheapest NSFV item. This ensured that a variety of NSFV items were included in the analysis, consistent with the regional recommendations for dietary variety [15]. The minimum daily cost for an individual to meet the NSFV recommendations was then converted to a monthly cost by multiplying it by the average number of days per month between October and December.

For each household in the HIES sample, a monthly household NSFV cost was determined by multiplying the monthly individual NSFV cost by the number of household occupants. As the NSFV recommendations [14] are population-wide with no separate recommendations for children, no adjustments were made for household occupant composition. The monthly household NSFV cost was then compared against the household’s monthly total expenditure (i.e. household budget) and food expenditure (i.e. food budget) to assess the affordability of meeting the NSFV intake recommendations.

The HIES data were also used to calculate the actual monthly NSFV expenditure for each household in the sample. Consistent with the inclusion criteria used in determining the minimum NSFV cost, the measure of household NSFV expenditure excluded imported NSFV, nuts and garnishes. Each household’s monthly NSFV expenditure was compared against their monthly NSFV cost to determine the adequacy of their NSFV expenditure. Adequate NSFV expenditure was defined as household NSFV expenditure ≥ 100% the household NSFV cost.

Summary statistics were generated and the results also disaggregated according to household size and total expenditure decile (a measure of household wealth). These analyses were performed for the entire sample of 578 households, thus including households with no recorded NSFV expenditure. The local currency (vatu) was converted to US dollars using the exchange rate at 15 November 2010: 1vatu = $0.011161 US [31].

## Results

### Fruit and vegetable costs

After excluding erroneous data and out-of-scope items, prices for 56 NSFV were available for analysis, comprising 21 market items and 35 supermarket items. When ranked on a price per 80 g edible serve basis, the first 19 of the cheapest 20 items were all from the central produce markets. Prices of the 10 cheapest NSFV per edible serve are provided in Table 1.

**Table 1:** **Cheapest 10 NSFV per edible serve in Port Vila during the December 2010 quarter**

**Non-starchy fruit or vegetable**	**Price (vatu)**	
	**Per kg**	**Per edible 80 g serve**
Pumpkin	75.02	8.57
Pamplemousse *(Citrus paradisi)*	55.54	8.89
Snake beans *(Vigna unguiculata subsp. sesquipedalis)*	116.71	9.82
Island cabbage *(Abelmoschus manihot)*	131.73	10.54
Pawpaw *(Carica papaya)*	82.40	10.63
Banana	88.94	11.12
Passion fruit	75.00	11.54
Carrot	132.25	11.89
Watercress	146.72	12.77
Chinese cabbage	142.27	12.93

The minimum daily cost for an individual to meet the recommended minimum intake of at least 400 g (five serves) of NSFV in Port Vila during the December 2010 CPI quarter was 48.46 vatu per day, or 1,486.24 vatu per month (approximately $16.60 US). This cost comprised an 80 g edible serve of pumpkin, pamplemousse, snake beans, island cabbage and pawpaw.

### Household expenditure

Means, relative standard errors (RSE) and standard deviations (SD) for Port Vila household’s total, food and NSFV expenditure are presented in Table 2. There was a high level of variability in the data, as indicated by the relatively large standard deviations.

**Table 2:** **Port Vila household total, food and NSFV expenditure**

**Monthly household expenditure**	**Minimum (vt)**	**Maximum (vt)**	**Mean (vt)**	**Standard deviation**	**RSE**
Total	12,111.27	556,902.20	102,747.38	75,064.22	5.09%
Food	3,228.96	406,022.19	42,067.45	33,151.67	5.34%
NSFV^a^	0.00	66,388.98	4,808.56	7,069.31	9.27%

vt, vatu.

NSFV, non-starchy fruit and vegetable.

RSE, relative standard error.

^a^includes households with no recorded fruit and vegetable expenditure.

The mean number of occupants in Port Vila households was 5.06 (range 1-19). As shown in Table 3, mean total monthly household expenditure was 102,747.38 vatu (approximately $1,146.76 US); with 45.1% (range 1.9 - 93.2%; SD 19.9%) of this spent on food, including 4.7% (range 0.00 - 43.4%; SD 5.1%) on NSFV. All forms of expenditure tended to increase with increasing household size, however differences were significant (P < 0.001) only for total and NSFV expenditure. The difference in the proportion of food expenditure spent on NSFV according to household size also approached significance (P = 0.069).

**Table 3:** **Total, food, and NSFV expenditure according to household occupant size**

		**Mean monthly household expenditure**					
**HH occs**	**n**	Total (vt)*	**Food (vt)**	NSFV (vt)*	**Food/ total**	**NSFV/ total**	**NSFV/ food†**
**1-2**	81	67,703.48	26,820.18	2,545.28	45.7%	3.7%	8.5%
**3-4**	189	91,117.03	37,014.00	4,192.99	47.1%	5.1%	10.9%
**5-6**	158	103,936.11	42,312.06	5,444.30	43.8%	5.0%	12.1%
**7-8**	102	124,800.55	53,929.81	5,769.03	43.7%	4.4%	10.0%
≥**9**	48	156,902.59	61,682.49	6,917.96	44.1%	4.8%	11.5%
**Total**	578	102,747.38	42,067.45	4,808.56	45.1%	4.7%	10.8%

HH occs, household occupants.

vt, vatu.

NSFV, non-starchy fruit and vegetable.

*P < 0.001; ANOVA for differences between household size.

† P < 0.069; ANOVA for differences between household size.

### Fruit and vegetable affordability

**Figure 1:**
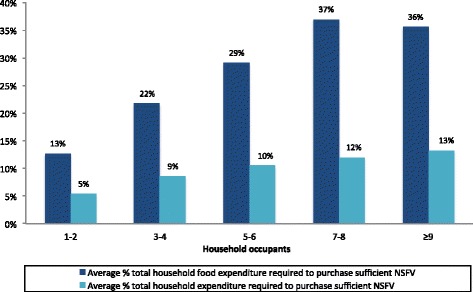
**NSFV affordability according to household size.**

**Figure 2:**
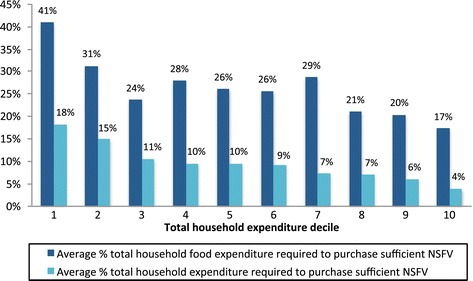
**NSFV affordability according to total household expenditure decile.**

Purchasing sufficient NSFV would account for an average of 9.6% (SD 6.4%) of a Port Vila household’s total budget and 26.3% (SD 25.8%) of their food budget. Greater proportions of the household budget would be required for larger or poorer households to purchase sufficient NSFV (Figures 1 and 2). This is to be expected, given these households’ respective greater NSFV requirements and limited economic capacity.

As Figure 2 illustrates, the wealthiest households, i.e. those in the tenth household expenditure decile, would need to allocate only 17.3% (SD 16.3%) of their food budget to NSFV to purchase recommended amounts, whereas the poorest households in the first expenditure decile would need to allocate 40.9% (SD 34.3%) of their food budget to NSFV to meet their household’s requirements.

### Adequacy of expenditure

**Figure 3:**
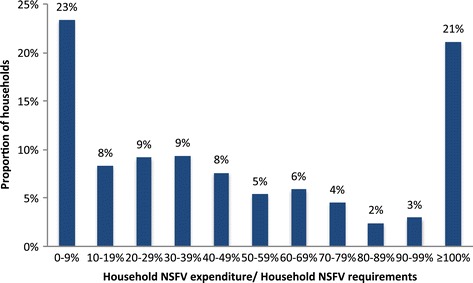
**Adequacy of household NSFV expenditure.**

Port Vila households spent an average of 72.3% of the expenditure required to purchase recommended amounts of NSFV for all household occupants. As shown in Figure 3, only 21.1% of Port Vila households purchased sufficient NSFV to meet the recommendations and almost one-quarter (23.4%) of households spent less than one-tenth of the cost of meeting their household’s NSFV requirements.

**Figure 4:**
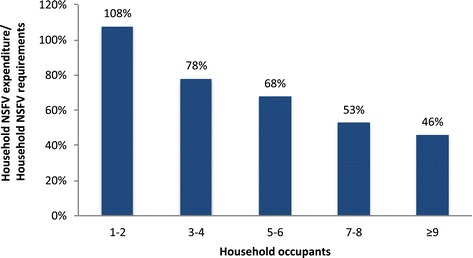
**Adequacy of expenditure according to household size.**

When adequacy of expenditure was analysed according to household size, only households with 1-2 occupants purchased sufficient NSFV. There was an inverse relationship between household size and adequacy of NSFV expenditure, with larger households having least adequate NSFV expenditure (Figure 4).

## Discussion

To assess the affordability of an item, it is necessary to understand the cost of the item and consider that in the context of an individual’s (or household’s) economic capacity to purchase that particular item. This study utilised secondary data analysis to calculate the cost of purchasing recommended amounts of NSFV in Port Vila, Vanuatu, and considered that in relation to a household’s total expenditure, a measure of economic capacity [32].

The relatively low RSEs for this study indicate that the data are of good quality and provide reliable estimates of true expenditure patterns. Approximately 80% of households in Port Vila did not spend enough on NSFV to meet the recommendations, even when the cheapest varieties were considered. This is higher than the estimate determined from the WHO STEPS survey [10] which reported that 62% of adults in Vanuatu consumed insufficient quantities of NSFV. However, the WHO STEPS survey sampled both rural and urban areas across Vanuatu, whereas the present study focused only on Port Vila, Vanuatu’s largest urban centre where population growth and urbanisation introduce unique challenges to food security and NSFV access and affordability [19,20]. The present study also considered the NSFV requirements of all household occupants; thereby capturing children for whom poor diets can have lifelong detrimental effects on growth and development [33]. Despite the different methodologies and results, both studies report largely inadequate NSFV intakes in Vanuatu when compared against the *minimum* recommendations for these foods.

To the best of our knowledge, NSFV cost and affordability has not been assessed in a comparable manner elsewhere in Pacific Island countries. The findings of this paper are consistent with international evidence which reports that insufficient NSFV intakes are common in low and middle-income countries, particularly amongst the poorest [16,34-36]. In order to purchase the recommended amount of NSFV for all household members, the poorest households in Port Vila would need to spend almost 20% of their total budget and more than 40% of their food budget on NSFV. This would leave little capacity to purchase other essential foods, as well as necessities such as fuel, electricity, school fees, transport and medical costs.

Overall, global food prices are becoming more expensive and increasingly volatile [37]. The poor are most affected by this situation, especially those in developing countries who may spend up to 75% of their income on food [37]. Countries reliant on food imports, which includes the majority of Pacific Islands, are particularly vulnerable to international food price increases and volatility [25].

High food costs can be a barrier for low-income households in making healthy food purchases [26,38,39] and can drive low-income farming households to sell their produce for a profit and purchase cheaper and less nutritious foods instead [39]. Low-income households tend to consume nutrient poor and energy dense diets, which are inconsistent with dietary guidelines and associated with increased risk of obesity, NCDs and malnutrition [26,38,39]. Households with lower expenditure on fruits and vegetables are also at greater risk of under-5 child mortality [40].

A reduction in population dietary energy density, which may be achieved through increased consumption of energy dilute fruits and vegetables, is a recognised public health strategy for obesity prevention and improving macronutrient intakes [14]. This may increase diet costs as an inverse relationship between a food’s energy density and its energy cost, i.e. cost per kilojoule, has been observed in various contexts including the United States and France [41], urban South Africa [42] and remote Australian Aboriginal communities [43]. However, the use of this metric has been questioned [44,45] and foods that are consistent with dietary guidelines have been reported to cost less than foods high in added sugars, added fats or sodium when compared on a cost per edible weight or cost per portion basis [46].

The results of this study indicate that improving the affordability and consumption of energy-dilute and nutrient-dense NSFV will be important in the prevention of NCDs and micronutrient deficiencies in Vanuatu. Research in Brazil has estimated that reducing fruit and vegetable prices by 20% would lead to an increase of approximately 16% of the proportion of these foods in the national diet [47].

One opportunity to improve NSFV affordability is through reducing wastage and increasing the efficiency of production and supply systems [48]. Approximately one-third of food produced for human consumption globally is wasted [48] and this can significantly reduce developing countries’ capacity to meet NSFV intake recommendations [39]. Due to their perishable nature and short shelf-life, fruits and vegetables are particularly vulnerable to wastage throughout the harvesting and distribution process [49,50]. Opportunities are available to support developing countries to improve their fruit and vegetable production processes [50], and these are worthy of consideration.

The findings from this study can be used to support targeted food and agricultural policy development in Vanuatu. Guidelines for the promotion of local produce in the Pacific Islands are available [25] and are based on a fruit and vegetable promotion program that was developed in the Federated States of Micronesia. In this program, multi-sectorial community-based promotion of local food production and consumption was found to be associated with an increased frequency of household consumption of fruits and local vegetables and an increased variety of local foods generally [51]. The present study has identified the cost per edible serve of locally produced NSFV, which will assist to promote the cheapest ways to meet NSFV recommendations in Vanuatu.

Secondary analysis of the Vanuatu HIES and CPI datasets provided the opportunity to explore the issue of NSFV cost and affordability in a cost-effective and efficient manner. Secondary analysis of household expenditure survey data has been estimated to be 75 times cheaper than conducting a 24-hour food recall survey [52]. The periodical nature of these data collections also offers the opportunity to monitor trends in NSFV cost and affordability over time and evaluate the impact of relevant health and agricultural policies. Options for improving the use of household expenditure surveys for nutrition analysis should be considered in future survey enumerations [53,54].

### Study limitations

This study used household NSFV expenditure data as a proxy measure for NSFV consumption. This type of data cannot account for food wastage at the household level, which may have resulted in an over-estimation of the proportion of households meeting the NSFV recommendations. However, food wastage is in developing countries is generally lower than that of developed countries, particularly at the household level [48] which may lessen the impact of this error.

Household food expenditure data also assumes an even distribution of foods amongst household members. This may not be the case, as gender differences exist in the amount of NSFV consumed by adults in Vanuatu, with men more likely than women to meet daily intake recommendations (42% compared to 35%) [10]. Consumption may also differ by age of household member [16]. Despite these limitations, estimates from household expenditure surveys compare well against 24-hour recalls for estimates of food consumption at the household level [55].

The food cost information in this study was obtained from the Vanuatu CPI. While the NSFV prices in the CPI collected were extensive, they are not exhaustive, as market food prices were only collected from the central market in Port Vila and smaller roadside markets were excluded. If produce at roadside markets was cheaper than the central markets, we may have underestimated the proportion of households purchasing recommended amounts of NSFV.

The CPI food prices were used to calculate the cost per edible serve of each NSFV item. As the Pacific Islands Food Composition Tables [56] does not contain information on the edible portions, US Food Composition Tables [30] were used to adjust food prices for the inedible portions. It is possible that people in Vanuatu prepare NSFV with more or less wastage than what is reported in the US Food Composition Tables, which may have also introduced potential error into the results of this study.

Another study limitation of this study is the use of household expenditure, rather than income, as a measure of household wealth. A valid and reliable measure of household income is difficult to obtain in developing countries due to widespread under-reporting and the often ad-hoc nature of wages in the informal work sector, as well as part-time or seasonal work opportunities [27,32]. The 2010 HIES household income data were also affected by respondent error [27]. Thus analysts prefer to use expenditure data in developing countries as an indicator of household wealth as it tends to smooth-out income fluctuations and is easier to measure [32]. In addition, errors may have been introduced in the expenditure data if the estimated monetary value participants assigned to the subsistence produce they consumed, and the amount they consumed, was under-reported [23,57]. However, this issue may be more relevant to rural areas where subsistence agriculture is more common [23].

The HIES was conducted between October and December 2010, and the corresponding CPI prices were selected to match the HIES enumeration period. Therefore this analysis may not represent annual NSFV cost and affordability in Port Vila, as food costs and households’ finances may be influenced by seasonal fluctuations in factors such as crop yields, employment opportunities, and household expenditure.

This study assessed the cost and affordability of meeting the WHO/FAO expert panel recommendations for fruits and vegetable intake [14], and therefore starchy crops such as sweet potato, yam, taro, potato, plantain banana and breadfruit were excluded from the analysis. This is justified as regional nutrition recommendations [15] group starchy crops with carbohydrate-based foods; estimates of the burden of disease attributable to low fruit and vegetable intake exclude starchy crops [58,59]; and other studies [16,34] that assess fruit and vegetable intakes, including the WHO STEPS survey [60] have excluded starchy crops. Including starchy crops in this analysis would require information on households’ dietary energy requirements, which was not available through the information collected in the HIES survey. We acknowledge that the exclusion of starchy crops in this analysis limits the ability to compare the results with studies that have classified fruit and vegetable items differently. Inconsistency in the definition of fruits and vegetables is a recognised issue affecting comparability of research in this field [34,59,61].

To be consistent with regional recommendations which promote consumption of local foods [15], imported NSFV items were excluded in this study. This is unlikely to significantly affect the results, as the CPI data indicated that these items were expensive relative to local produce, and the Vanuatu HIES survey [27] reported that these items were generally not commonly purchased foods, accounting for only 3.1% and 0.4% of respective fruit and vegetable expenditures in urban areas.

Lastly, as there are no separate recommendations for children’s NSFV consumption, the population-wide recommendation of 400 g of NSFV per day [14] was applied for all household members. Thirty two percent of the urban population in Vanuatu is younger than 15 years [2]. If children’s recommended intakes are lower than adults, this study may have over-estimated the proportion of households with insufficient NSFV expenditure, and the relationship between NSFV affordability and household size. Establishing and applying children’s NSFV requirements would assist to improve the accuracy of the results of this study.

## Conclusions

Expenditure data on NSFV in the capital and main urban centre of Vanuatu in 2010 indicates that most households were not purchasing the recommended number of NSFV serves to meet the needs of all household members. NSFV affordability declined with household size, indicating that larger households were at greatest risk of inadequate intakes. The cost of purchasing the recommended amount of NSFV would account for almost 20% of the poorest households’ total budget and over 40% of their food budget, even considering the cheapest varieties available. This analysis indicates that NSFV affordability and consumption in urban areas is an important consideration in the implementation of agricultural and health policies in Vanuatu.

### Epilogue

This analysis was conducted before the devastating effects of cyclone Pam hit the island nation of Vanuatu on 13 March 2015. The cyclone destroyed most of the banana and root crops, as well as all island cabbage plants and other leafy vegetables. Fruit trees were stripped and most coconuts were felled. Regeneration efforts are hampered by loss of food and seed stocks. Food security is further threatened following the loss of small livestock, including chickens and pigs and the destruction of fisheries infrastructure, including canoes, small boats, and fishing gear. These losses have been suffered by a large proportion of Vanuatu’s population.

The impact of the cyclone will further impact the access, cost and affordability of NSFV and other nutritious foods in Vanuatu and further monitoring of food availability, access and affordability in Port Vila is necessary to provide current information on this topic.
